# Supplementary material for: HIVprotI: an integrated web based platform for prediction and design of HIV proteins inhibitors
Source: J Cheminform. 2018 Mar 9;10:12. doi: 10.1186/s13321-018-0266-y (PMC5845081; doi:10.1186/s13321-018-0266-y)
Supplement: Supplementary file 2 — Additional file 2. Source code of HIVProtI web server. [file 13321_2018_266_MOESM2_ESM.zip › HIVprotI_Source-code/hivprotI/weka-3-6-6/doc/weka/core/PropertyPath.PathElement.html]

PropertyPath.PathElement


---


|  |  |  |
| --- | --- | --- |
| **Overview** \| **Package** \| **Class** \| **Tree** \| **Deprecated** \| **Index** \| **Help** \| **Weka's home** | |  |
| **PREV CLASS**   **NEXT CLASS** | **FRAMES**    **NO FRAMES**     **All Classes** |  |
| SUMMARY: NESTED \| FIELD \| CONSTR \| METHOD | DETAIL: FIELD \| CONSTR \| METHOD |  |


---


## weka.core Class PropertyPath.PathElement

```
java.lang.Object
  weka.core.PropertyPath.PathElement
```

**All Implemented Interfaces:**: java.lang.Cloneable, RevisionHandler

**Enclosing class:**: PropertyPath

---

``` public static class PropertyPath.PathElement extends java.lang.Object implements java.lang.Cloneable, RevisionHandler ```

Represents a single element of a property path

**Version:**
:   $Revision: 4742 $

**Author:**
:   fracpete (fracpete at waikato dot ac dot nz)

---

**Constructor Summary**

`PropertyPath.PathElement(java.lang.String property)`
  
          initializes the path element with the given property


| **Method Summary** | |
| --- | --- |
| `java.lang.Object` | `clone()`             returns a clone of the current object |
| `int` | `getIndex()`             returns the index of the property, -1 if the property is not an index-based one |
| `java.lang.String` | `getName()`             returns the name of the property |
| `java.lang.String` | `getRevision()`             Returns the revision string. |
| `boolean` | `hasIndex()`             returns whether the property is an index-based one |
| `java.lang.String` | `toString()`             returns the element once again as string |

**Methods inherited from class java.lang.Object**

`equals, getClass, hashCode, notify, notifyAll, wait, wait, wait`

**Constructor Detail**

### PropertyPath.PathElement

```
public PropertyPath.PathElement(java.lang.String property)
```

:   initializes the path element with the given property

    **Parameters:**: `property` - the property to initialize with


**Method Detail**

### clone

```
public java.lang.Object clone()
```

:   returns a clone of the current object

    :   **Overrides:**: `clone` in class `java.lang.Object`
    :   **Returns:**: the clone of the current state

---


### getName

```
public java.lang.String getName()
```

:   returns the name of the property

    :   **Returns:**: the name of the property

---


### hasIndex

```
public boolean hasIndex()
```

:   returns whether the property is an index-based one

    :   **Returns:**: true if the property has an index

---


### getIndex

```
public int getIndex()
```

:   returns the index of the property, -1 if the property is not an
    index-based one

    :   **Returns:**: the index of the property

---


### toString

```
public java.lang.String toString()
```

:   returns the element once again as string

    :   **Overrides:**: `toString` in class `java.lang.Object`
    :   **Returns:**: the property as string

---


### getRevision

```
public java.lang.String getRevision()
```

:   Returns the revision string.

    :   **Specified by:**: `getRevision` in interface `RevisionHandler`
    :   **Returns:**: the revision


---


|  |  |  |
| --- | --- | --- |
| **Overview** \| **Package** \| **Class** \| **Tree** \| **Deprecated** \| **Index** \| **Help** \| **Weka's home** | |  |
| **PREV CLASS**   **NEXT CLASS** | **FRAMES**    **NO FRAMES**     **All Classes** |  |
| SUMMARY: NESTED \| FIELD \| CONSTR \| METHOD | DETAIL: FIELD \| CONSTR \| METHOD |  |


---
